# Thermosonication Combined with Natural Antimicrobial Nisin: A Potential Technique Ensuring Microbiological Safety and Improving the Quality Parameters of Orange Juice

**DOI:** 10.3390/foods10081851

**Published:** 2021-08-11

**Authors:** Qinyu Zhao, Quyu Yuan, Chenxu Gao, Xiaoyang Wang, Bihe Zhu, Jiaqi Wang, Xiangyu Sun, Tingting Ma

**Affiliations:** College of Food Science and Engineering, College of Enology, Northwest A&F University, Xianyang 712100, China; zqy1004@nwafu.edu.cn (Q.Z.); y23933@nwafu.edu.cn (Q.Y.); 2018013406@nwafu.edu.cn (C.G.); 18821712077@163.com (X.W.); zbhxinong@nwafu.edu.cn (B.Z.); hello-wjq@nwafu.edu.cn (J.W.); sunxiangyu@nwafu.edu.cn (X.S.)

**Keywords:** orange juice, nisin-assisted thermosonication, microbial and enzyme inactivation, sensory quality, bioactive properties

## Abstract

Currently, thermal pasteurisation (TP) remains the most widely applied technique for commercial orange juice preservation; however, a high temperature causes adverse effects on the quality attributes of orange juice. In order to explore a novel non-thermal sterilization method for orange juice, the impacts of thermosonication combined with nisin (TSN) and TP treatments on the quality attributes including microbial and enzyme inactivation and the physicochemical, nutritional, functional, and sensory qualities of orange juice were studied. Both TP and TSN treatments achieved desirable bactericidal and enzyme inactivation effects, and nisin had a significant synergistic lethal effect on aerobic bacteria in orange juice (*p* < 0.05). Additionally, TSN treatment significantly improved the color attributes of orange juice and well maintained its physicochemical properties and sensory quality. More importantly, TSN treatment significantly increased the total polyphenols content (TPC) and total carotenoids (TC) by 10.03% and 20.10%, increased the ORAC and DPPH by 51.10% and 10.58%, and the contents of total flavonoids and ascorbic acid were largely retained. Correlation analysis of antioxidant activity showed that the ORAC and scavenging ability of DPPH radicals of orange juice are mainly attributed to TC and TPC. These findings indicate that TSN shows great potential application value, which could guarantee the microbiological safety and improve the quality attributes of orange juice.

## 1. Introduction

Orange juice has gained worldwide popularity owing to its high nutritional value, attractive color, and distinctive sweet and sour flavor [1]. It is rich in a variety of nutritional and bioactive phytochemicals, including a high concentration of vitamin C, folate, polyphenols, flavonoids, carotenoid, and limonoid [2,3,4]. Epidemiological studies have shown that the consumption of orange juice is beneficial to human health, e.g., by enhancing the antioxidant activity of blood serum [5] and lowering lipid peroxidation [6], improving risk factors associated with cardiovascular disease [7], and improving the cognitive function of the elderly [8]. According to the United States Department of Agriculture in 2016, the annual consumption of orange juice is about 1.8 billion liters globally [9].

Sterilization is an important unit operation in the production of orange juice. Currently, conventional thermal pasteurisation (TP) remains the most widely applied technique for commercial orange juice preservation and is considered the safest and most cost-effective way to inactivate microorganisms and enzymes [10]. However, a high temperature may cause the deterioration of the color and flavor of orange juice [11,12,13] and the loss of heat-sensitive nutrients and functional substances [14,15], which might impair the functional properties and sensory attributes of the final product, thus making it unattractive to the consumer. Thus, it is necessary to explore a new non-thermal sterilization method which can not only effectively inactivate microorganisms and enzymes, but also maintain the optimal sensory attributes and the nutritional and functional characteristics of orange juice.

Ultrasound (US) processing is one of the emerging non-thermal sterilization technologies recently being applied in liquid food. Its mechanism of inactivated microorganisms and enzymes is generally attributed to the combination of mechanical effects (e.g., cavitation, bubble rupture, and mechanical shear force) and chemical effects (e.g., the formation of free radicals and the decomposition of water vapor in collapsing bubbles) [13,16]. Recently, US has been intensively investigated as a means to preserve fruit and vegetable juice; however, some researchers have shown that using US alone in some cases may not be very efficient for the inactivation of some types of microorganisms and enzymes in juices [16,17,18]; meanwhile, US is combined with other sterilization techniques, for example, mild heat treatment [i.e., thermosonication (TS)] [16,19], ultraviolet treatment [20], a pulsed electric field [21], gassing [22], and TS with antibacterial agents [23,24]. These showed high application potential in the preservation of various juices. Various US-combined sterilization techniques not only showed a synergistic effect in the inactivation of microorganisms and enzymes, but also maintained the sensory, physicochemical, and nutritional attributes of juices. Among them, due to the low costs and a simple operation, combine use of TS and TS treatment with an antibacterial agent (for example, nisin) have been a wide concern [16,24,25]. Mild heat treatment, which uses a lower heating temperature during thermal processing (usually lower than 60 °C), could bring down, even remove the loss of heat-sensitive nutrient substances and bioactive phytochemicals, thus prevented the deterioration caused by high thermal temperatures to juice quality [19,24]. Nisin is a heat-stable antimicrobial peptide produced by strains of Lactococcus lactis subsp. lactis. It is hydrolyzed into amino acids by a protease in the digestive tract after consumption and does not produce resistance or allergic reactions [26]. It is a safe antimicrobial peptide for food preservation, which was recognized by the WHO (World Health Organization). The bactericidal mechanisms of nisin include inhibiting the cell wall biosynthesis, leading to ATP hydrolysis and ion leakage, membrane pore formation and disruption of the pH equilibrium and the proton motive force, and eventually to cell death [24,26].

Orange juice is very popular in the word, and significantly contributes to the daily fruit consumption in most countries; thus, the commercial importance of orange juice is self-evident. In recent years, in order to improve the overall quality of commercial orange juice, many non-thermal processing technologies for the sterilization process of orange juice have been widely reported, including high-pressure processing [27], pulsed electric fields [2], US processing [13], TS processing [28], cold plasma [29], ozone processing [29], dimethyl dicarbonate [30], and the ultrasound-assisted supercritical CO_2_ system [22]. However, the research on the combination of TS and nisin is rarely reported. Therefore, the purpose of this study was to (i) compare the germicidal efficacy of TS, TS combined with nisin (TSN), and TP treatments on microorganisms in orange juice, (ii) compare the impacts of TSN and TP treatments on overall juice quality (enzyme inactivation, physicochemical, nutritional and functional and sensory qualities), and (iii) analyze the correlation between the antioxidant capacity, color characteristics, and functional substances of orange juice. The research aims to provide a theoretical basis and technical support for the high-quality orange juice produce.

## 2. Materials and Methods

### 2.1. Orange Juice Preparation

Fresh navel oranges grown in Jiangxi province (China) were used in the study. The oranges were washed and peeled, cut into four pieces, and pressed mechanically by a juice extractor. The pressed orange juice was filtered through a sterilized double-layered muslin cloth, homogenized at 4 °C and 150 bar, transferred into a sterile food-grade container, and stored at 4 °C until further treatment.

### 2.2. Chemicals and Reagents

All the standards, including 1,1-diphenyl-2-picrylhydrazyl (DPPH), gallic acid, 6-hydroxy-2,5,7,8-tetramethylchromane-2-carboxylic acid (Trolox), catechol, and Folin-Ciocalteu reagent were purchased from Sigma-Aldrich (St. Louis, MO, USA). All media, including the lauryl sulfate peptone broth, Bengal red medium, plate counting medium, and bright green lactose bile broth, were purchased from Hope Bio-Technology Co., Ltd. (Qingdao, China).

### 2.3. Preparation of Nisin Solution

Referring to the method of Ma et al. (2020) [24] with minor modifications, 0.5 g of commercial nisin Z (900 IU/mg) was dissolved in 20 mL of sterile water, then filtered through a 0.22-μm inorganic membrane to remove the microorganisms, and kept at 4 °C for the further experiments.

### 2.4. TS, TSN, and CTS Treatment

Fresh orange juices were treated with TS, TSN, and TP respectively, with fresh orange juice (no sterilization treatment) as the control. TS treatment [24,25] was conducted by a ATPIO-1000D built-in probe ultrasound device (Xianou Corporation of Nanjing, Jiangsu, China), operating with a frequency of 20–25 kHz, a maximum electrical power input of 1000 W, and a horn microtip diameter of 6 mm. Juice samples of 45 mL were subjected to a double-wall cylindrical vessel, in which water was circulated with a digital thermostatic bath (XODC-0515-II, Nanjing Xianou, Nanjing, China) to control the TS temperature at a constant 50 °C. The power was adjusted to 70% of the maximum power, and the processing time was 10 min, with the pulse duration time set as 2 s on and 3 s off. For the TSN treatment, adding 360 µL of nisin solution to samples to reach a final concentration of 200 ppm, then it was placed in a double-walled cylindrical vessel for the same operation as the TS treatment. For the TP treatment, orange juice samples of 45 mL were pasteurized at 80 °C for 10 min using an electro-thermostatic water bath [13]. Each treatment was carried out at least in triplicate (Table S1).

### 2.5. Microbiological Assay

The viable cells of natural microorganisms in the orange juice samples were measured based on the National Food Hygiene Standard of China. The total bacterial count (TBC), Escherichia coli, and mold were detected according to GB 4789.2-2016, GB 4789.3-2016, and GB 4789.3-2016, respectively. The specific experimental operation refers to the method of Ma et al. (2020) [31]. Results are expressed as the log CFU/mL and log MPN/mL.

### 2.6. Determination of the Activities of the Polyphenol Oxidase (PPO), Peroxidase (POD), and Pectin Methylesterase (PME)

PPO and POD was extracted by dissolving 4% (*w*/*v*) polyvinyl pyrrolidone (PVPP), 1% (*v*/*v*) Triton x-100, and 1 M NaCl in a 0.2 M phosphate buffer (pH = 6.5). Orange juice and enzyme extracts (1:1, *w*/*w*) were mixed, extracted at 4 °C for 2 h, and centrifuged at 10,000 g/min for 20 min at 4 °C. The supernatant was taken to determine the PPO and POD activities. The enzyme activities were measured based on the method of Ma et al. (2020) [24].

PME was extracted by mixing juice samples and NaCl (8.8% *w*/*v*) at a ratio of 4.5:15 (*w*/*v*) and centrifuged at 15,000× *g* for 20 min at 4 °C. The supernatant was collected to determine the PME. PME activities were measured exactly according to the method of Muthukumarappan et al. (2009) [32].

### 2.7. Physicochemical Indexes

The total soluble solids (TSS) of the orange juice were measured by a PAL-1 digital Abbe Refractometer (ATAGO Co., Tokyo, Japan). The preparation of the titratable acid (TA) extract and the measurement of TA using the acid–base titration method were based on China National Standard GB/T12456-2008 [33]. The pH value was evaluated by a PHS-3E pH meter (Shanghai Leici Co. Ltd., Shanghai, China). The juice viscosity was determined using an NDJ-5S rotary viscometer (Jinan Jingtian Co. Ltd., Jinan, China).

### 2.8. Functional Indices and Antioxidant Activity

The ascorbic acid (AA) of juice samples was determined by the 2,6-dichlorindophenol method, the specific experimental operation according to the literature of Ma et al. (2020) [31]. The total polyphenol content (TPC) and total flavonoid content (TFC) were measured by the Folin–Ciocalteu colorimetric method (Ma et al., 2019) [34] and the aluminum chloride colorimetric assay (Aadil et al., 2013) [35], and results are expressed as milligrams of gallic acid equivalents (GAE) per liter (mg GAE/L) and milligrams of catechol equivalents per liter (mg CTE/L), respectively. The content of total carotenoids (TC) was measured based on the method of Abid et al. (2020) [36], and results are expressed as milligrams of β-carotene equivalents per liter.

The antioxidant capacity of the orange juice with different treatments was measured by the DPPH free radical scavenging ability, the ferric ion reducing antioxidant power (FRAP), and the oxygen radical antioxidant capacity (ORAC) method based on previous reports [34,37]. Results are expressed as millimoles of trolox equivalents per liter (mM TE/L).

### 2.9. Sensory Evaluation

#### 2.9.1. Electronic Nose (E-Nose) Assay

The aroma profiles of the orange juice samples were obtained with a portable PEN 3 E-nose (Airsense Analytics, Schwerin, Germany) containing 10 metal oxide semiconductors. Each sensor has a certain degree of affinity for specific volatile compounds. Specific descriptions of the sensor and the specific operation steps are found in our previous work [24,31]. Each sample was measured at least 10 times.

#### 2.9.2. Color Determination

The color characteristics of the orange juice samples were measured by an X-Rite ci7600 colorimeter in reflection mode. In the CIELab scale, L* represents lightness, a* represents greenness to redness, b* represents yellowness to blueness, and the total color difference (ΔE*), hue (H°), chroma (C*), L*, a*, and b* were automatically determined by the colorimeter or calculated by its own software.

#### 2.9.3. Artificial Sensory Evaluation

Previously trained panelists Potential consumers (18 men and 18 women, from 20 to 45 years old) participated in the sensory evaluation. These panelists were previously trained with the basic knowledge of sensory evaluation, so that they could do a better sensory assessment. The overall quality of the juice samples was evaluated by 5 different attributes (appearance, color, smell odor, sweet and sour suitability, and overall acceptance) using a 9-point hedonic scale a quantitative descriptive analysis with 100 score (Tables S2 and S3). The specific experimental operation is in accordance with our previous work [31].

### 2.10. Statistical Analysis

Data analysis was performed using Excel 2016, SPSS 23, RStudio-1.1.463, and Origin 9.1. The experimental results are expressed as the means ± standard deviations (SD) of three replicates for each treatment.

## 3. Results and Discussion

### 3.1. Microbial Inactivation of Different Treatments

**Figure 1:**
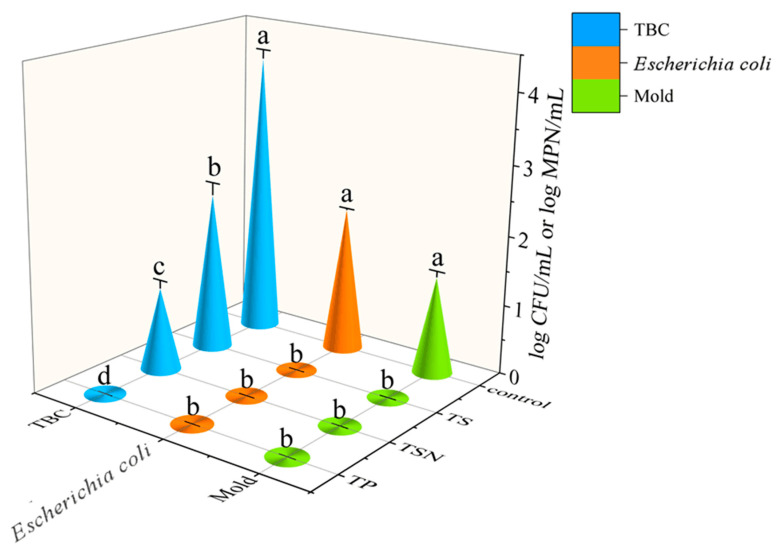
Lethal effects of different sterilization treatments on the microorganisms in orange juice. The *Escherichia coli* results are expressed as log MPN/mL, and the other results are expressed as log CFU/mL.

As shown in Figure 1, the TBC, Escherichia coli, and mold in the control group were 4.04 log CFU/mL, 2.07 log MPN/mL, and 1.4 log cfu/mL, respectively. After TP, TS, and TSN treatment, the TBC decreased to 0, 2.26, and 1.18 log CFU/mL, respectively, and no Escherichia coli or mold were detected in the orange juice samples, which indicated that all three treatments showed a strong inactivation effect on Escherichia coli and mold in the orange juice. Undoubtedly, TP was still the most efficacious and thorough sterilization method. Meanwhile, the effect of TS on TBC was limited, while TSN significantly enhanced the bactericidal effect (*p* < 0.05), indicating that nisin and TS had synergistic inactivation effects on aerobic bacteria, which is consistent with Ma et al.’s (2020) and Liao et al.’s (2018) results. The effect of nisin on the goal bacteria occurs by inhibiting the cell wall biosynthesis and membrane pore formation and disrupting the pH equilibrium and the proton motive force, leading to ATP hydrolysis, ion leakage, and finally, to cell death [24,26]. Compared with another new non thermal processing pulsed electric field (PEF) treatment, under 24.8 kV/cm, 60 pulses, 169 μs treatment time, 53.8 °C PEF treatment, apple juice cannot be stored at room temperature, because the PEF treatment cannot provide enough energy to inactivate microorganisms under this condition. TSN can make orange juice meet the microbiological safety standards and it is a good and gentle sterilization method [38].

Based on the Chinese national standard GB7101-2015, when the TBC is less than 2 log CFU/mL, the number of Escherichia coli is less than 1 MPN/mL, and the juice is considered safe to drink. Therefore, in this study, in addition to TS treatment, both TSN and CTS treatments could ensure the microbiological safety of the orange juice (Figure 1). TSN and CTS treatments were selected for further research.

### 3.2. Residual Enzyme Activities of Different Treatments

**Figure 2:**
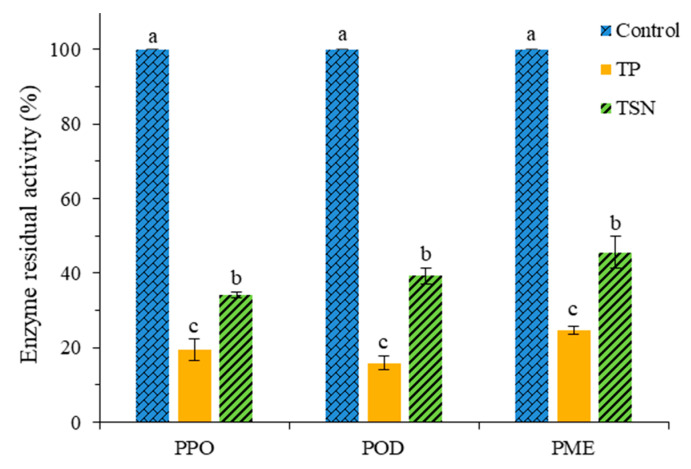
Passivation effects of different sterilization treatments on the enzyme activity of orange juice.

The residual activities of PPO, POD, and PME with different sterilization treatments are shown in Figure 2. After TP treatment, the remainder activities of PPO, POD, and PME were 19.41%, 15.83%, and 24.75%, respectively. After TSN treatment, the residual activities of these three enzymes were 34.19%, 39.30%, and 45.60%, respectively. Obviously, the enzyme inactivation effect of TSN treatment was weaker than that of the TP treatment (*p* < 0.05), but compared with the control group, TSN still showed a desired inactivation effect, reducing the PPO, POD, and PME activities by 65.81%, 60.70%, and 54.40%, respectively, significantly reducing the activity of three endogenous enzymes in orange juice (*p* < 0.05).Therefore, the two treatments could effectively control the enzymatic browning of the orange juice and maintain its cloud stability. Compared with high isostatic pressure (HIP) treatment, Patrícia Martins de Oliveira and others have shown that the use of HIP to process mango–carrot juice mixed juice can effectively inactivate polyphenol oxidase (PPO), but it will increase peroxidase (POD) activity. It is not conducive to the long-term preservation of juice [39].

### 3.3. Physicochemical Properties of Different Treatments

**Figure 3:**
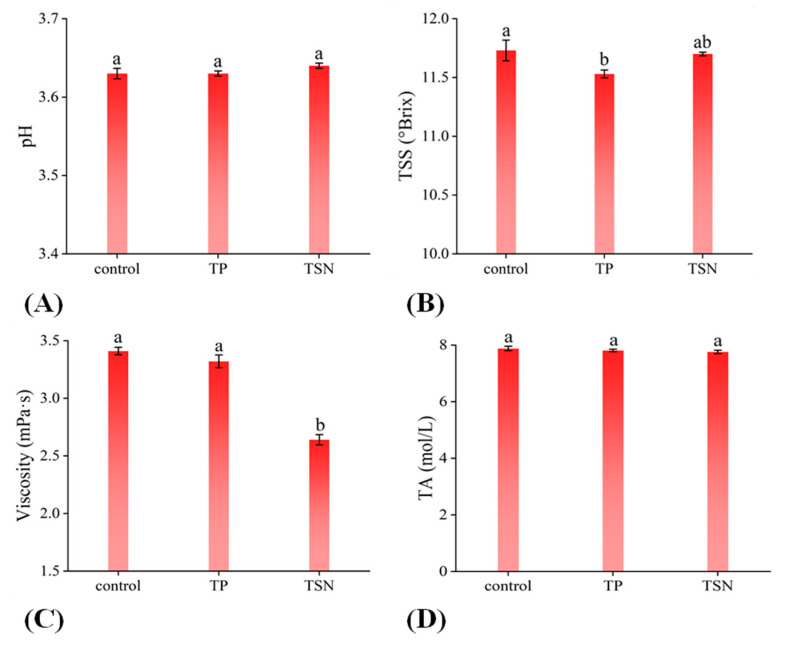
Effects of different sterilization treatments on the physicochemical properties of orange juice. (**A**) TSS; (**B**) pH; (**C**) viscosity; and (**D**) TA.

The effects of TP and TSN on pH, TSS, TA, and viscosity are shown in Figure 3A–D. Compared with the control group, no significant change was observed in the pH, TSS, and TA of the TSN group (*p* > 0.05), which indicated that TSN did not alter pH, TSS, or TA of the orange juice, which is consistent with the findings of Liao et al. (2018) [25] and Walkling-Ribeiro et al. (2009) [40]. Previous studies have shown that TSN had no influence on the viscosity of grape juice [24] and green juice (celery stalk, apple, cucumber, parsley) [41]; however, in this study, TSN treatment significantly reduced the viscosity of the orange juice, and this was mainly due to the difference in operating parameters and juice matrix. In addition, TP treatment significantly reduced the TSS of the orange juice (*p* < 0.05). Ma et al. also obtained similar results with grape juice [24]. In the research on grape juice, it was also found that PEF treatment will not affect its pH, TSS, and acidity [42].

### 3.4. Functional Indices of Different Treatments

#### 3.4.1. AA and TFC

**Figure 4:**
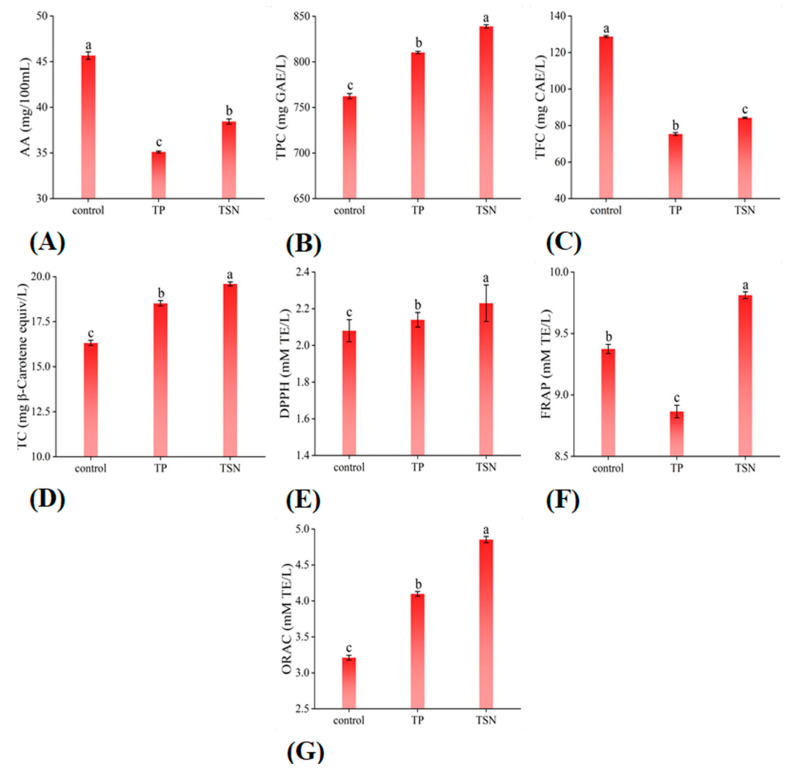
Effects of different sterilization treatments on the functional indicators of orange juice. (**A**) AA; (**B**) TPC; (**C**) TFC; (**D**) TC; (**E**) FRAP assays; (**F**) DPPH scavenging activity; (**G**) ORAC assays.

The influence of TP and TSN on the nutritional and functional indicators is shown in Figure 4A–G. Orange juice is well known as an appreciable source of AA; unfortunately, AA in juice is unstable and is easily affected by environmental factors such as temperature, pH, dissolved oxygen, and metal ions [43,44]; thus, AA is easily lost during juice processing. As shown in Figure 4A, compared with the control group, the AA content decreased by 23.10% and 15.83% after TP and TSN treatment, respectively. Thus, the different sterilization treatments all caused a certain loss of AA, but compared with TP, TSN could significantly slow down the loss of AA (*p* < 0.05) and retain the AA to the greatest extent. Similar results were also reported by Liao et al. (2018) [25], who found an 87.42% retention of AA exposed to TSN treatment at 52 °C for 30 min. This was mainly due to the mild temperature (<60 °C) applied during the TSN processing. Ultrasonic cavitation reduced the level of dissolved oxygen in the orange juice, thus inhibiting the degradation and loss of AA [19].

As shown in Figure 4C, the effect of TP and TSN treatments on the TFC of orange juice was similar to that of AA (Figure 4A). TFC suffered 41.45% and 34.55% of losses after TP and TSN treatment, respectively. Nevertheless, the TFC retention rate of TSN-treated orange juice was much higher than that of TP-treated samples (*p* < 0.05). A recent study on the effects of different sterilization treatments on the quality attributes of grape juice also reached the same conclusion [24].

#### 3.4.2. TPC and TC

Polyphenols are important antioxidant components in plants, and their content is particularly abundant in citrus fruits. As shown in Figure 4B, both TP and TSN treatments increased the TPC in orange juice, while TSN showed the highest effect, as it added TPC value from 762.41 mg GAE/L to 838.89 mg GAE/L, with an increase of 10.03% (*p* < 0.05). Similar results were also reported in TSN-treated grape juice [24], TS-treated hog plum juice [45], and TS-treated star fruit juice [46].

Similarly, both TP and TSN treatments significantly increased the TC in orange juice (*p* < 0.05); it increased by 13.48% and 20.10%, respectively, after TP and TSN treatment (Figure 4D). Due to the lipophilic nature of carotenoids and their specific localization in plant tissues, appropriate processing can promote the release and dissolution of carotenoids [47].

Figure 4A–D shows that TSN treatment significantly increased the functional components in orange juice, as TPC increased by 10.03%, and TC increased by 20.10%. This is principally because the cavitation effect during TSN treatment increases the mechanical disruption of the plant cell wall, so as to promote the dissolution and extraction efficiency of the nutrients and functional components of juice [45]. Meanwhile, TSN treatment significantly reduced the related oxidase activities and dissolved oxygen levels in juice; thus, it can be employed as a preservation technique for orange juice processing with a high retention of AA and TFC.

#### 3.4.3. Antioxidant Activity

FRAP, ORAC, and DPPH are considered to be the most widely used in antioxidant activity measuring, as they can reflect the antioxidant capacity from different aspects. As shown in Figure 4E, the FRAP values of the control group, the TP group, and the TSN group were 9.37, 8.86, and 9.81 mM TE/L, respectively. TP treatment significantly reduced the FRAP of orange juice, whereas TSN treatment greatly enhanced the FRAP value. Additionally, both the TP and TSN treatments significantly increased the ORAC and DPPH values of orange juice compared with the control group (Figure 4F,G), but the TSN-treated juice showed the highest ORAC (4.85 mM TE/L) and DPPH (2.23 mM TE/L) values, which were 51.10% and 10.58% higher than in the control group and were 15.46% and 4.70% higher than in the TP group. A large number of published studies have also confirmed that US-related treatments, such as US, TS, and TSN, could significantly enhance the antioxidant activity of juice [17,24,45,46]. This is mainly because the ultrasonic process promotes the extraction and release of antioxidants, such as polyphenols and carotenoids [24,45].

Figure 4A–G shows that TSN treatment significantly increased or highly retained the contents of nutrients and functional substances in orange juice and significantly enhanced its antioxidant activity in three different systems. The enhancement of antioxidant content and antioxidant activity are important manifestations of the nutritional value of orange juice. Therefore, TSN treatment, as a new non-thermal sterilization method, can significantly improve the nutritional quality of orange juice.

### 3.5. Correlation Analysis of Antioxidant Activity

**Figure 5:**
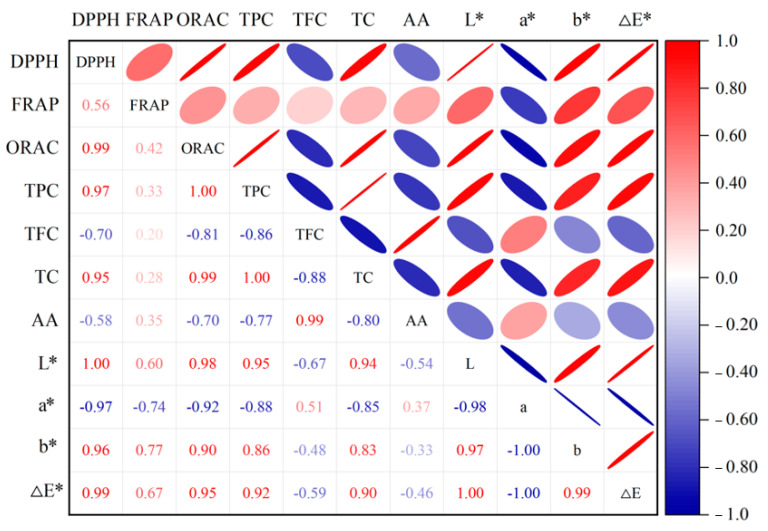
Correlation between the color attributes, antioxidants and antioxidant capacity of orange juice.

Previous studies have proven that several antioxidants such as ascorbic acid (AA), polyphenols, flavonoids, and anthocyanins might contribute to the antioxidant activity of various juice, and the color properties might also be related to the antioxidant activity [24,48,49]. In order to clarify the potential phytochemicals that contribute to the antioxidant capacity of orange juice, the correlations between the antioxidant capacity, color characteristics, and functional substances of orange juice were also analyzed, and the results are shown in Figure 5.

As shown in Figure 5, the closer the absolute value of the correlation coefficient is to 1, the closer the shape of the graph is to an ellipse. The closer the correlation coefficient is to 1, the redder the color is. On the contrary, the closer the correlation coefficient is to −1, the bluer the color. Thus, the indicators that are highly correlated with ORAC and DPPH are TC (RORAC = 0.99, RDPPH = 0.95), TPC (RORAC = 0.95, RDPPH = 0.88), L* (RORAC = 0.98, RDPPH = 1.00), b* (RORAC = 0.90, RDPPH = 0.96), and ΔE* (RORAC = 0.95, RDPPH = 0.99), which indicates that the antioxidant substances TPC and TC in orange juice and the color attributes L*, b*, and ΔE* make a greater contribution to the DPPH free radical scavenging ability and ORAC. Similar results were also observed by Zhao et al. (2018) [50], who found significant positive correlations between antioxidant activity and both TC and TFC in Lycium barbarum juice. However, in previous studies, Wang et al. (2019) [48] found that the antioxidant activity of strawberry juice was highly correlated with TPC, AA, and TFC; Ma et al. (2020) [24] reported that TPC plays a prominent role in the antioxidant activity of grape juice. Due to the complexity of the antioxidant activity of liquid foods, it includes different lipids and water-soluble compounds. Therefore, the correlation between the different methods used to determine antioxidant capacity mainly depends on the food matrix [51]. Furthermore, no significant correlation was observed between FRAP and the indicators measured in this study, which may be because the FRAP of orange juice was associated with multiple indicators, none of which was a major contributor.

### 3.6. Effects of Different Sterilization Treatments on the Sensory Quality

#### 3.6.1. Color Analysis

Juice color might affect consumers’ buying decisions and affect the flavor sensory characteristics of juice [52]. Hence, the color of orange juice after different sterilization treatments is also an important factor in measuring its quality. Table 1 displays the color parameters changes of orange juice after different sterilization treatments.

As Table 1 shows, compared with the control group, the a*, b*, H°, and C* values of orange juice with TP treatment did not change significantly (*p* > 0.05), and the ΔE* value is less than 2 CIELAB units. Fernández-Vázquez et al. (2013) [53] proposed that only when the ΔE* value between two orange juice samples is greater than 2.8 CIELAB units can consumers distinguish color. Therefore, juice color is basically unchanged after TP treatment. However, TSN treatment significantly increased the L*, b*, H°, and C* values of orange juice (*p* < 0.05), which indicated that the lightness, yellowness, and color saturation of orange juice increased significantly after TSN treatment, and this change caters to consumers’ psychological expectations concerning the ideal color of orange juice [53]. Studies have shown that the color characteristics of most commercial orange juice are as follows: a L* value from 61 to 66, an H° value from 79 to 93, and a C* value from 42 to 60 [52,53]. This color range can be accepted by most consumers and is considered an ideal orange juice color. From Table 1, it is obvious that the L* and C* values of TSN-treated orange juice are closer to the ideal color. In general, TSN treatment significantly improved the color attributes and had a positive impact on the quality of the orange juice.

#### 3.6.2. Artificial Sensory Evaluation

**Figure 6:**
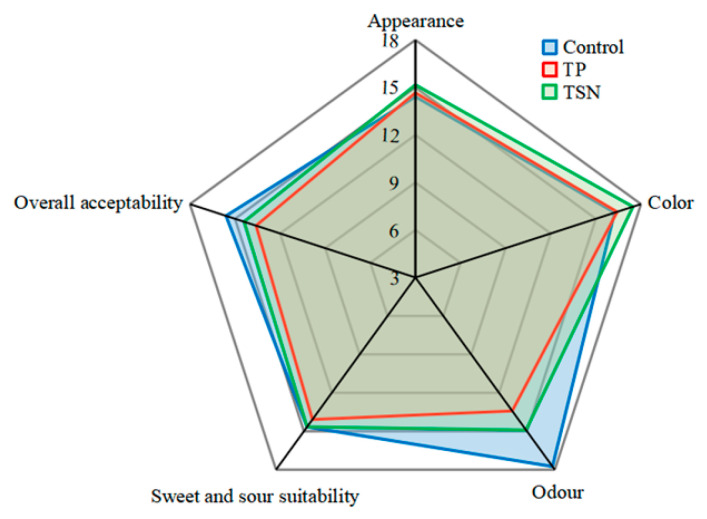
Spider plot of artificial sensory evaluation of orange juice under different sterilization treatments.

Sensory properties are very important for the consumers’ acceptance or rejection of food. The sensory evaluation including color, odor, appearance, sweet and sour suitability, and overall acceptability of orange juice is illustrated in Figure 6. Among the three groups, the overall acceptability of samples in control was highest, mainly because of its optimal sweet and sour suitability and odor attribute. Conversely, the scores of the various sensory attributes of orange juice in the TP group were relatively low, thus its overall acceptability is the lowest. During the sensory experiment, it was found that the TP-treated orange juice had an obvious sour taste. Our previous research also proved that TP treatment could increase the acidity of grape juice [24]. The TSN-treated orange juice had the highest score of all color attributes, which is consistent with the result of the color analysis in Section 3.6.1. In addition, the odor attribute of the orange juice altered significantly after different sterilization treatments. The TP-treated orange juice had the lowest odor attribute score, and this is mainly because the high temperature during the TP process negatively affects its aroma profile [10,27]. The odor score of the TSN-treated juice was significantly higher than that treated by TP.

In general, TSN treatment significantly improved the color attributes of the orange juice. There was no significant difference between TSN and the control group in terms of the appearance and sweet and sour suitability, and the odor score of the TSN group was relatively high. Thus, the overall acceptability of the TSN group was significantly higher than that of the TP group. TSN treatment can well maintain the sensory quality of orange juice.

#### 3.6.3. E-Nose Analysis

**Figure 7:**
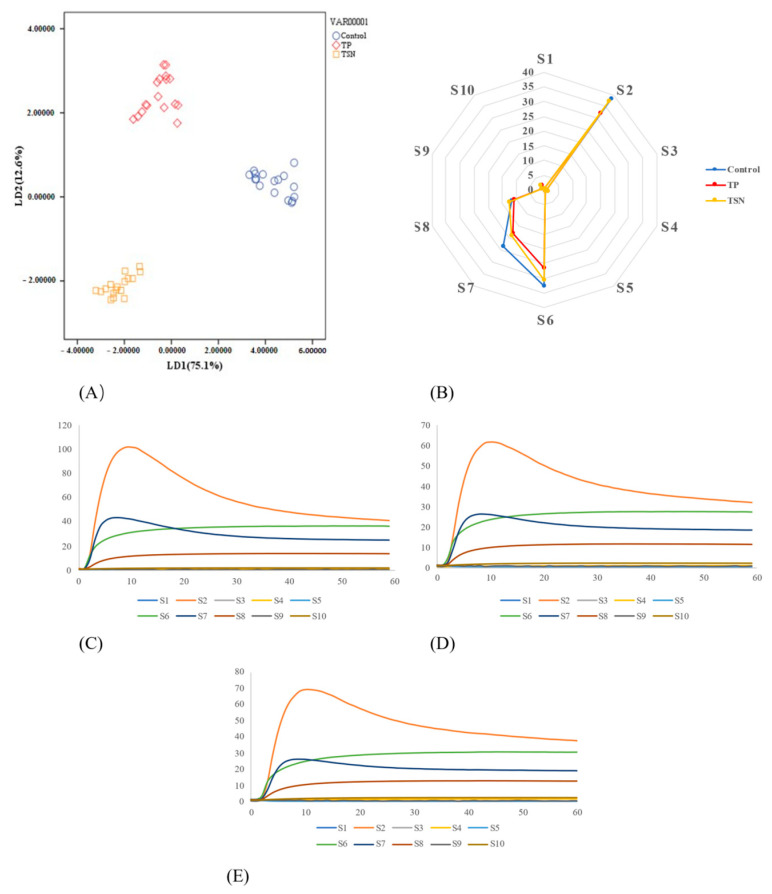
E-nose assay of orange juice after different sterilization treatments. (**A**) LDA results of orange juice under different sterilization treatments; (**B**) radar chart of E-nose response data of orange juice after different sterilization treatments; sensor responses recorded using ten sensors in the E-nose for (**C**) control group; (**D**) CTS treatment; and (**E**) TSN treatment.

Figure 7C–E shows the records of the control, TP, and TSN groups. The LDA linear discriminant method was used to analyze the average stable signal of the ten sensor of E-nose at the last 5 s [24], and the results are shown in Figure 7A. It can be seen in Figure 7A that the two discriminant functions could explain 87.7% of the whole variance, of which LD1 explained 75.1%, and LD2 explained 12.6%. E-nose using the LDA model could clearly distinguish the different samples after different treatments.

In addition, a radar chart was drawn based on the response data of different sensors for the orange juice’s odor profile. Figure 7B shows that the orange juice samples of the three groups only responded to Sensors S2 (sensitive to nitrogen oxides), S6 (sensitive to methane, in a broad range), and S7 (sensitive to many sulfur organic compounds and terpenes. The response values of the control group were all the highest, while the response values of each sensor reduced significantly after TP treatment. After TSN treatment, the response value of Sensor S7 decreased significantly, while the response values of Sensors S2 and S6 were not significant different from the control group. These indicated that both TSN and TP treatments caused varying degrees of odor substance loss in the orange juice. In general, TSN showed a close odor characteristics compared to control, which was in keeping with the artificial sensory evaluation.

## 4. Conclusions

The effects of TSN and TP on the overall quality of orange juice were systematically studied in this paper. The results indicate that both TSN and TP could guarantee the microbial safety firstly. Although the enzyme inactivation effect of TSN was weaker than that of TP, it also exhibited a desirable effect. In terms of the nutritional and functional characteristics, TSN treatment significantly increased the content of the functional components in orange juice. It increased the TPC by 10.03% and the TC by 20.10%, and the antioxidant capacity, e.g., the ORAC and DPPH values, increased by 50% and 10%, respectively. Meanwhile, the TAC and TFC were highly retained. In terms of sensory quality, TSN treatment significantly improved the color attributes of the orange juice, and the overall acceptability of the TSN-treated juice was significantly higher than that of the TP-treated juice. In addition, TSN treatment basically did not change the physicochemical indicators of the orange juice, while TP treatment significantly reduced the TSS.

In summary, based on ensuring the microbial safety firstly, TSN can not only well maintain the physicochemical indictors and sensory quality of orange juice, but TSN could also greatly improve the functional and nutritional characteristics of orange juice. Thus, TSN can be considered a novel non-thermal technique with the potential to assist producers to produce high-quality juice; thus, it8 has high commercial application prospects. In addition, in future studies, in order to explore the wider application of this method in other fruit and vegetable juice matrix, it is necessary to add an amount of specific microorganisms to measure its effectiveness.

## Figures and Tables

**Table 1:** The color parameters of control, TSN, and TP treatment juices.

Processing Method	Color					
	L*	a*	b*	ΔE	H°	C*
Control	51.60 ± 0.03 ^c^	−2.02 ± 0.01 ^a^	29.96 ± 0.04 ^b^	0.00 ± 0.00 ^c^	93.86 ± 0.03 ^b^	30.03 ± 0.04 ^b^
TP	52.85 ± 0.45 ^b^	−2.18 ± 0.17 ^a^	30.44 ± 0.77 ^b^	1.46 ± 0.55 ^b^	94.11 ± 0.43 ^b^	30.52 ± 0.75 ^b^
TSN	55.07 ± 0.16 ^a^	−2.92 ± 0.03 ^b^	33.39 ± 0.12 ^a^	4.96 ± 0.18 ^a^	95.00 ± 0.06 ^a^	33.51 ± 0.12 ^a^

Note: Different letters represent significant difference (*p* < 0.05) from each other in the same column.

## Data Availability

The datasets generated for this study are available on request to the corresponding author.

## Supplementary Materials

The following are available online at https://www.mdpi.com/article/10.3390/foods10081851/s1, Table S1: The treatment conditions of different treatments, Table S2: The standard table for sensory rating of orange juice, Table S3: The table of sample scoring.
