# Supplementary material for: Thermosonication Combined with Natural Antimicrobial Nisin: A Potential Technique Ensuring Microbiological Safety and Improving the Quality Parameters of Orange Juice
Source: Foods. 2021 Aug 11;10(8):1851. doi: 10.3390/foods10081851 (PMC8393855; doi:10.3390/foods10081851)
Supplement: Supplementary file 1 [file foods-10-01851-s001.zip › foods-1308757-SI.pdf]

Table S1 The treatment conditions of different treatments

Treatment groups	Required instrument	Treatment conditions	Nisin addition concentration /ppm
TS	ultrasound device (ATPIO-1000D）	700W, with a pulse duration of 2 s on and 3 s off, 50℃, 10 min	0
TSN	ultrasound device (ATPIO-1000D）	700W, with a pulse duration of 2 s on and 3 s off, 50℃ 10 min	200
TP	electro-thermostatic water bath	80℃ 10 min	0

Table S2 The standard table for sensory rating of orange juice

Score	Appearance	Color	Odour	Sweet and sour suitability	Overall acceptability
16~20	The system is uniform, without precipitation and stratification, and the juice is clear	It is orange yellow with good luster	Orange juice has a strong aroma	Sour, sweet and delicious, sweet aftertaste	I'd love to accept it
11~15	Slightly turbid, slightly precipitated or slightly stratified	It is slightly yellowish brown and shiny	Orange juice has medium aroma	Slightly out of balance between acidity and sweetness	Relatively acceptable
6~10	It is turbid, with a small amount of precipitation or pulp particles	Yellowish brown, poor gloss	Orange juice has a light aroma	Slightly sour or slightly sweet	Basically unacceptable
1~5	There is more stratification or turbidity	It is dark yellowish brown and dull	Basically no orange juice aroma	Too sour or too sweet	Totally unacceptable

Table S3 The table of sample scoring

No.	Appearance	Color	Odour	Sweet and sour suitability	Overall acceptability
1					
2					
3					
4					
5					
